# Case report: A suspected case of chronic pulmonary sparganosis characterized by migrating cavities and tunnel sign

**DOI:** 10.3389/fmed.2024.1453043

**Published:** 2024-09-19

**Authors:** Zhongfeng Niu, Lujiao Chen, Yanhua Zhang, Li Zhao

**Affiliations:** ^1^Department of Radiology, Sir Run Run Shaw Hospital, Zhejiang University School of Medicine, Hangzhou, China; ^2^Department of Radiology, Shaoxing People’s Hospital, Zhejiang, China

**Keywords:** pulmonary sparganosis, migrating cavities, tunnel sign, computed tomography, case report

## Abstract

A 20-year-old male patient with a 15-month history of recurrent cough and hemoptysis presented at our hospital with suspected pulmonary sparganosis. Computed Tomography (CT) revealed migratory and variable lesions ranging from patchy shadows to nodular and cavernous foci. Additionally, the location and morphology of the cavities changed rapidly. The patient’s peripheral blood eosinophil count remained within the normal range throughout the course of the infection, and antibiotics (moxifloxacin) alleviated the symptoms. At the early stage of admission, there was a slight increase in neutrophil and basophil counts. Initial treatment with a standard dose of praziquantel led to a significant improvement in symptoms, but the symptoms soon relapsed. However, doubling the dose 4 months later eventually cured the disease. The migratory nature of the CT lesion and the presence of tunnel signs were key to diagnosing a parasitic infection. The variability and rapid changes in the lesion further facilitated the differentiation of the disease, which rarely manifests as a granulomatous cavity.

## 1 Introduction

Pulmonary sparganosis is a zoonotic parasitic disease caused by *Spirometra* species larvae and infection in humans, who serve as incidental secondary hosts (1). Human sparganosis typically affects the subcutaneous tissue, ocular regions, whereas visceral organs, including the lungs, are rarely impacted (2–4). A nonspecific clinical presentation leads to delayed diagnosis and misdiagnosis of pulmonary sparganosis (5). Tunnel signs in the pulmonary parenchyma have been rarely reported in the literature (6), and cavernous lesions on chest CT have not been previously described in detail. In this report, we present a suspected case of the patient who experienced recurrent cough and hemoptysis for 15 months, which was visually characterized by migrating cavities and tunnel signs on CT.

## 2 Case report

**Figure 1:**
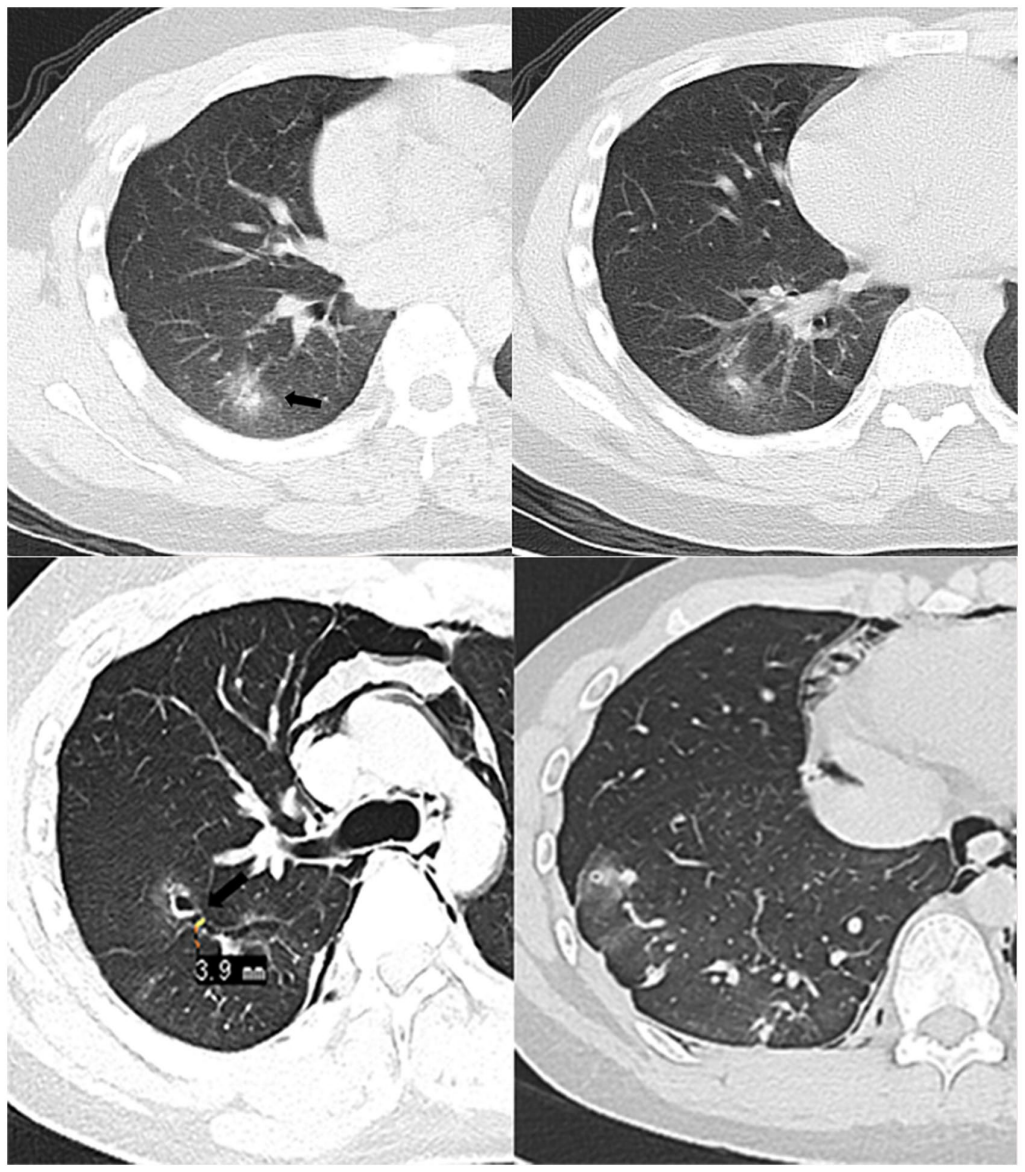
In March 2020 (Top row), the patient was seen for the first time. CT showed patchy nodules in the posterior basal segment of the right lower lung, with blurred edges and slightly higher-density arrows in the center. 2020.06 (Bottom row) Chest CT lung window, MRP oblique cross-sectional reconstruction. Pleural tunnel sign across the right oblique fissure (approximately 3.9 mm wide), the tunnel communicates with the cavitary lesion in the right upper lobe (arrow), accompanied by pneumothorax and pneumomediastinum.

In April 2020, a 20-year-old male college student presented to our respiratory medicine outpatient clinic, complaining of recurrent cough and hemoptysis for 5 months. These symptoms first appeared in November 2019 without an obvious cause. The patient coughed with a small amount of white mucus but did not have fever, wheezing, or cyanosis. Hemoptysis occurred at night, producing approximately 10 mL of bright red sputum. The patient did not experience shortness of breath or chest pain at that time, and therefore, he did not seek further medical attention. However, recurrent cough continued, and hemoptysis occurred more than 10 times a day, with varying amounts of bloody sputum ranging from 1 to 5 mL. Amid persistent symptoms, he was hospitalized in March 2020, and pulmonary CT showed a patchy ground-glass shadow in the right lower lung (dorsal segment, outer, and posterior basal segments), potentially indicating an infection (Figure 1). T-SPOT.TB test and Tuberculosis (TB) smear tests were negative. Hematological analysis revealed a white blood cell (WBC) count at 10.0 × 10^9^/L (normal range, 3.5–9.5 × 10^9^/L), a C-reactive protein (CRP) level at 13.0 mg/L (normal range, 0.0–5.0 mg/L) and eosinophil count at 0.07 × 10^9^/L (normal range, 0.02–0.52 × 10^9^/L), eosinophil percentage at 0.5% (normal range, 0.4–8.0%). Treatment with the antibiotics moxifloxacin alleviated the cough symptoms, and the patient continued oral antibiotic treatment after discharge. However, by April, the hemoptysis worsened to the state before hospitalization.

The patient had undergone an appendicitis operation 7 years earlier. On physical examination upon admission to our hospital, he was taking 19 breaths per minute, maintained a body temperature of 37.0°C and a heart rate of 87 bpm, and had normal blood pressure of 117/61 mmHg. Coarse breath sounds were heard in both lungs, without dry or wet rales. Chest CT revealed bilateral lower lung cord-like opacities and nodular shadows involving the upper right lung and both lower lungs, with a small lesion. Strip-like shadows connected lesions in the ipsilateral lung. Bronchoscopy findings were unremarkable, as acid-fast staining of the sampling brush, fungal test, and lavage fluid culture returned negative results. The glucan test results were <5.0 pg/mL, and all laboratory examinations, including biochemistry, blood routine, coagulation function, urine and stool routine, tumor markers, preoperative immunity, and ultrasensitive C-reactive protein, were normal. Infectious disease was highly suspected based on the multi-lesion CT imaging presentation and previous antibiotic treatment. However, there was a lack of pathogenic bacterial evidence or infection classification, so the physician empirically prescribed oral moxifloxacin at a dose of 400 mg/day. The hemoptysis disappeared, and the patient was discharged after 3 days of phlegm treatment.

In June 2020, the patient was admitted to the emergency department with hemoptysis and chest pain. Emergency chest CT revealed a small bilateral pneumothorax and extensive neck and mediastinal pneumatization. Multiple thin-walled cavities surrounded by non-uniform hazy rims were observed in both lungs, most of which connected through a tunnel structure. Figure 1 shows the tunnel sign crossing the oblique fissure of the right lung. Routine hematological tests were negative, and the CRP level was slightly elevated at 13 mg/L, which was inconsistent with a fungal or viral infection. Based on these imaging and clinical presentations, the radiologist suspected parasitic infection, and the patient’s serum was sent to a third-party testing company (Wuhan Kindstar Diagnostics Co., Ltd.) for the antibody test against *Sparganum* and other helminths (*Schistosoma japonicum*, *Paragonimus westermani Cysticerucus*, *Trichinella spiralis*, *Clonorchis sinessinensis*, and *Echinococcus* sp.) by multiple-dot ELISA. Given the positive result for *Sparganum* parasite, the patient was sent for a CT-guided biopsy of the right lower lobe. Pathological examination confirmed alveolar epithelial hyperplasia, foam cell aggregation in the alveolar lumen, iron-containing hematoxylin deposition, and a small amount of eosinophilic infiltration. The patient had lived long time in Xiangshan, Ningbo, Zhejiang Province, China, and then was admitted to Jinhua, Zhejiang Province, during the rest of the study period. Zhejiang is an endemic area of human sparganosis. Upon further inquiry, the patient stated that he had swum in a local reservoir 3 months before symptom onset and had accidentally ingested water from the reservoir. The patient had no history of raising or contacting pets, no overseas travel history, rarely consumed or handled raw seafood. Therefore, a diagnosis of pulmonary sparganosis was highly likely. Brain MRI and contrast-enhanced abdominal CT revealed no signs of parasitic infection in the examined body regions. Our local Center for Disease Control recommended the antiparasitic drug praziquantel. The patient received twice-daily dosing (four tablets per occasion, 200 mg/tablet) and underwent two courses of the medication regimen, with each course lasting for 3 days. The symptoms improved significantly after treatment but did not disappear completely.

**Figure 2:**
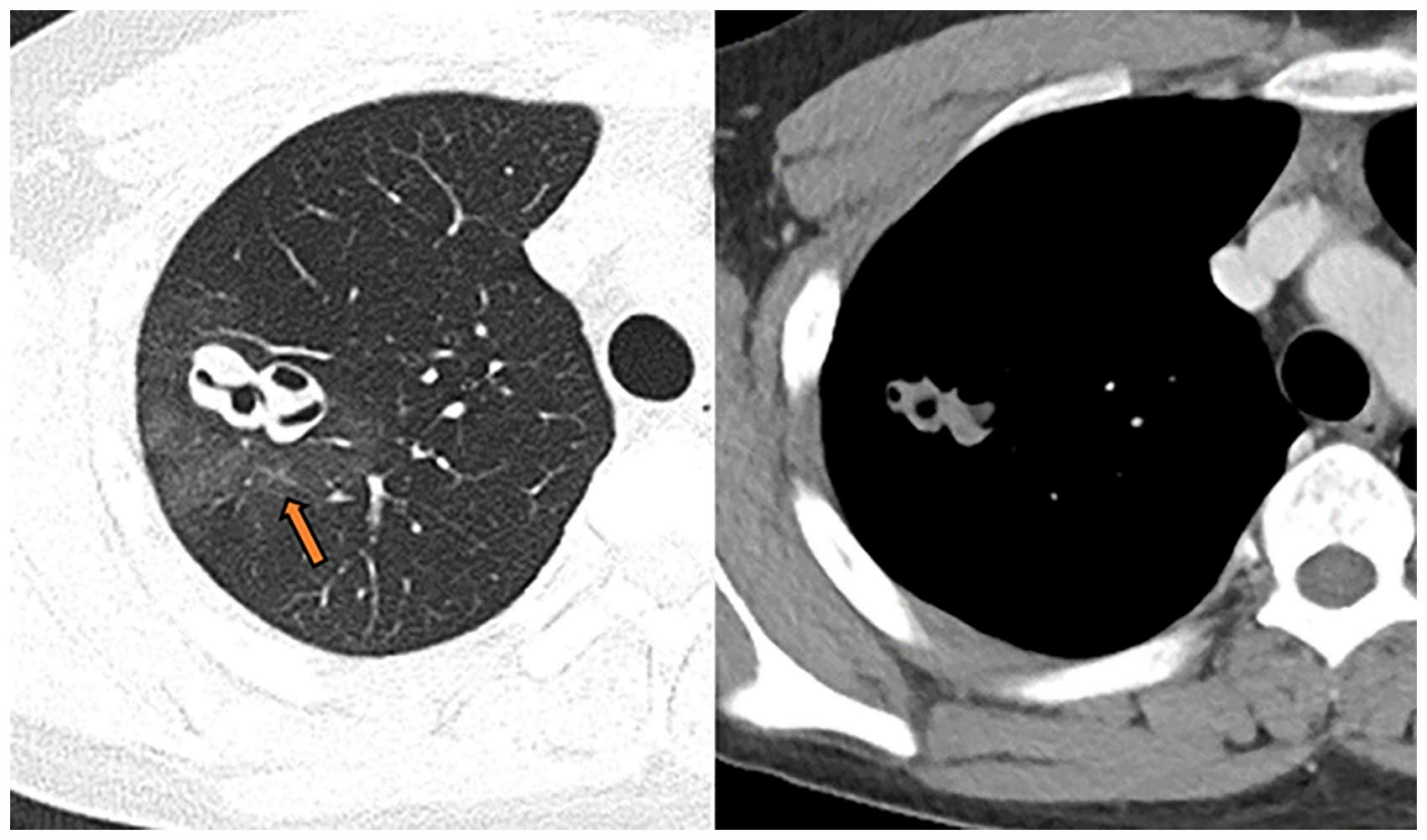
July 15, 2020, the wall of the right upper lung cavity punctured for the second time; it was surrounded by non-uniform hazy rim (pathology confirmed as intra-alveolar blood accumulation, arrow), the solid part was slightly enhanced, and the CT value increased from 56HU to 63HU on plain scan.

**Figure 3:**
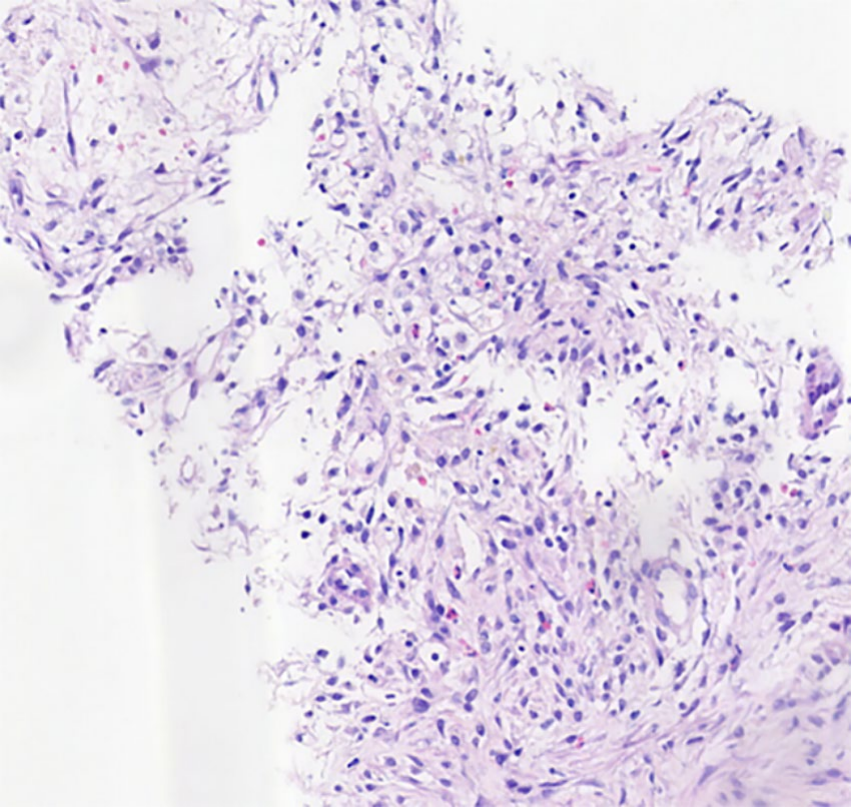
HE*100 lung tissue is accompanied by focal hemorrhage, necrosis, and fibrous tissue hyperplasia, hemosiderin-containing cell accumulation in the alveolar cavity, and a small amount of eosinophil infiltration.

In July 2020, the patient experienced recurrent cough and hemoptysis 1 week after the discontinuation of praziquantel therapy, along with chest tightness after activity and vague pain in the left posterior back on deep breathing. Chest CT revealed a new cavernous lesion with uneven wall thickness in the right upper lung, surrounded by a non-uniform hazy rim (Figure 2). Most cavities observed on the most recent chest CT scan had disappeared in June, leaving only one large cavity with a thin-walled cystic cavity that developed fibrosis in the surrounding par. Due to the relapse after antiparasitic treatment (before the puncture, we arranged for a chest CT enhancement examination), we re-evaluated our initial diagnosis of pulmonary sparganosis by performing another puncture biopsy of the new cavernous lesion. Figure 3 shows a small piece of lung tissue with focal hemorrhage, necrosis, fibrous tissue hyperplasia, aggregation of iron-containing hematoxylin in the alveolar cavity, and a small amount of eosinophil infiltrate. Given these inconclusive findings, the patient was again treated with moxifloxacin and was discharged with symptom improvement.

The patient was re-visited at another hospital in October 2020 for shortness of breath and chest pain, and a large right pleural effusion was observed on CT. Bronchoscopic lavage fluid did not yield any culture of pathogenic microorganisms, and lavage fluid metagenomic next-generation sequencing (mNGS) only detected oral/respiratory colonizing bacteria and *coccidioides*, which are commonly present in normal environments. The patient refused pleural fluid extraction or further testing. Lacking specific evidence of pathogenic microbial infections, we returned to the possibility conclusion of pulmonary sparganosis. The praziquantel dose was doubled and administered in combination with moxifloxacin. Subsequently, the lung lesions and pleural effusion gradually resolved, and the clinical symptoms steadily improved, eventually disappearing after 2 months. At the last imaging follow-up in October 2021, the lesions in both lungs were completely absorbed, leaving only a few fibrous foci in the lower lungs and a smooth, thin-walled air sac in the right upper lung. The patient was followed up by telephone until October 2022, with no recurrence of symptoms or signs of infection at other sites.

## 3 Discussion

Human sparganosis is a food-borne zoonosis caused by the spargana of various diphyllobothriid tapeworms of the genus *Spirometra*. Humans are accidental hosts, while dogs, cats, and other mammals serve as definitive hosts. Humans can acquire parasitic diseases by consuming frog meat or ingesting infested water. The larval stage of *Spirometra mansoni* poses significant health risks and can cause varying disease severity, depending on where the larvae migrate and reside in the human body. *Sparganum* infection is widely distributed in China, with the highest occurrence in Guangdong Province. Currently, there are no reports of large-scale epidemics or clustering. With the improvement of people’s living standards, food-borne parasitic diseases occur occasionally. In China, cases of gnathostomiasis caused by fish, which can cause systemic larva migrans, and sparganosis caused by eating snakes or frogs have been sporadically reported (7, 8). The patient lived in an endemic area on the east coast of mainland China (1).

Respiratory sparganosis can involve the pleura, bronchi, lung parenchyma, and pulmonary vasculature (6–9), and intrapulmonary lesions are often misdiagnosed as lung cancer or intrapulmonary infections (10). Elevated eosinophil counts in peripheral blood or body fluids typically suggest a diagnosis. However, in this case, elevated eosinophil counts in the peripheral blood were not observed at multiple admissions, which hindered our diagnostic confidence. A previous study reported that 25% of patients with pulmonary sparganosis did not show eosinoophilia (5). Since the patient had normal or slightly elevated hematological findings (including WBC, CRP, PCT, basophils) on several examinations, anti-infective treatment was administered empirically during the first two admissions based on the clinical manifestations and CT imaging findings. Radiologists raised the possibility of a parasitic infection, considering the migrating tunnel signs and granulomatous cavities. Nevertheless, a subsequent biopsy failed to provide a definitive diagnosis, although it ruled out tumors and conventional infectious diseases. Our suspicion of pulmonary sparganosis (3 months after the first visit and 7 months after the onset of symptoms) was further supported by a positive *sparganum* antibody and the patient’s self-declared exposure to reservoir water. After the patient experienced a relapse following praziquantel therapy, we performed mNGS with bronchoscopic lavage fluid to exclude the possibility of pathogenic bacterial infection and finally suspected parasitic infection. The effectiveness of the antiparasitic treatment after doubling the praziquantel dose further reinforces our diagnosis of pulmonary sparganosis. Other parasites such as *Anisakis* sp. and *Gnathostoma* sp. can also cause pulmonary migrating lesions. These parasites present as mass-like shadows in the lungs, similar to the early imaging manifestations in this case (11–13). Although those parasites were not included in the multiple-dot ELISA test performed by the commercial company, we considered our diagnosis of sparganosis for this case based on the patient’s residence, travel history, dietary habits and the specific antibody-positive by dot ELISA.

**Figure 4:**
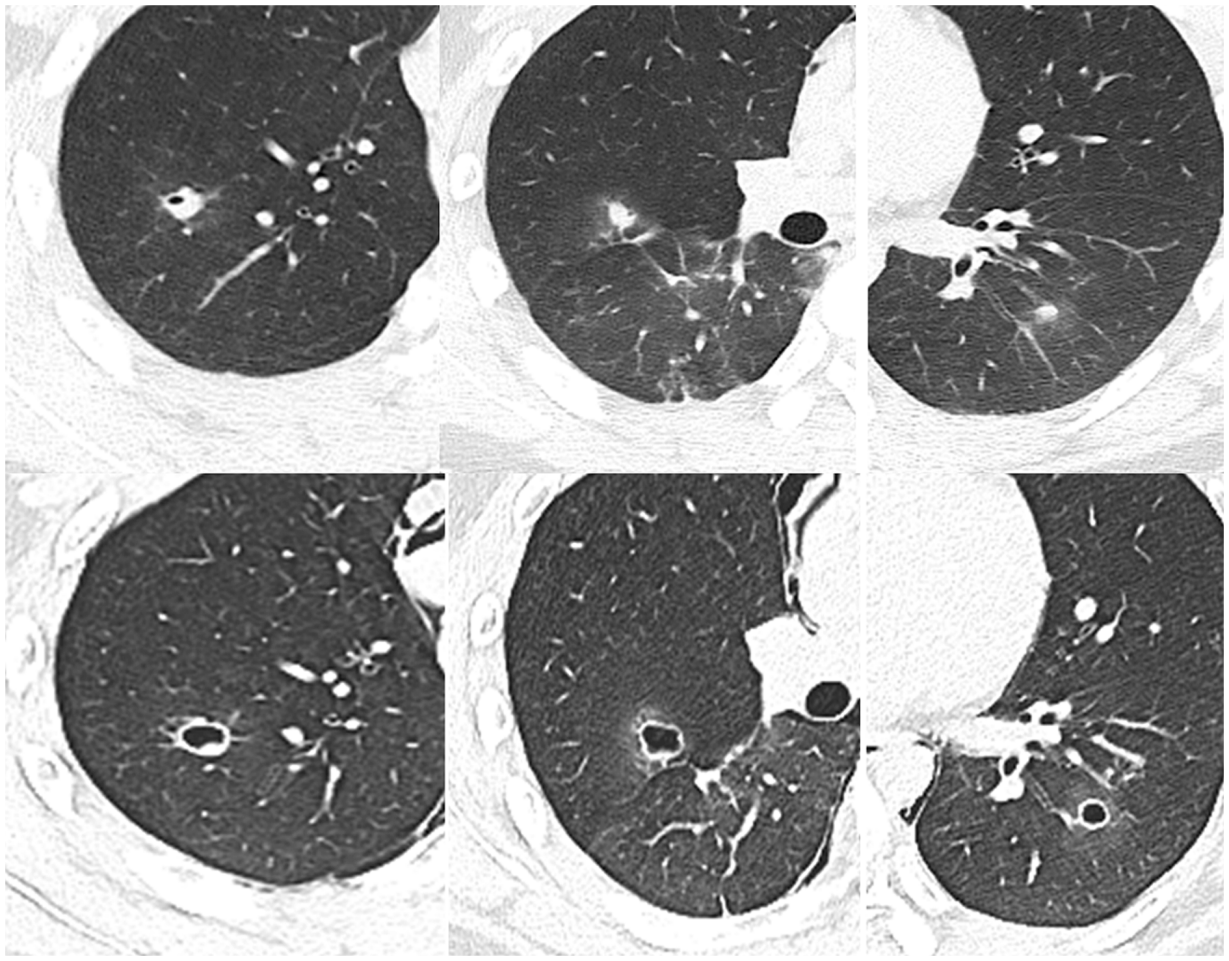
2020.07.13 (Top row) Admission CT shows pneumothorax and pneumomediastinum, multiple cavities in the lungs, and syncope (the tunnel shadow that appeared in June is now a cord shadow, arrow). 2020.07.15 (Bottom row) Chest CT enhanced scan: the shape of the lesions changes rapidly, with uniform thin-walled cavities.

Sparganosis infection of the lung parenchyma often appears in both lower lungs and can manifest as a variety of morphological patterns, including patchy, nodular, and mass lesions (14). The presence of migrating cavities and tunnel signs in the current case contributed to the suspected diagnosis of this disease (6). The cavities scattered throughout the lung parenchyma had uniformly thin walls and were surrounded by alveoli with accumulated blood. In particular, one cavernous lesion presented with irregular tortuosity without necrosis in the cyst wall and exhibited uniform enhancement. Serial CT examinations yielded the following findings: (1) Seven months after symptom onset, multiple cavernous lesions of varying sizes appeared, and intraalveolar blood accumulation was observed in the surrounding region (Figure 2). Such imaging behavior corresponds to the clinical manifestation of recurrent hemoptysis and has not been previously reported. (2) The tunnel sign that crossed the right lung oblique fissure suggested mechanical penetration of the lung and oblique cleft pleura and potentially explained the clinical presentation of chest pain, pneumothorax, mediastinal pneumothorax, and pleural effusion. (3) Between the non-contrast CT on July 13th and contrast-enhanced CT on July 15th, the cavitary lesions experienced significant changes in size and morphology, possibly reflecting rapid shrinkage of the original cavities and the formation of new cavities due to parasite movement (Figure 4). (4) Almost all lesions were completely absorbed after treatment except for the complex cavity in the right upper lung, which shrank to form a thin-walled pulmonary air sac with a small amount of fibrosis.

Pulmonary sparganosis is primarily treated with internal medicine; therefore, surgical and biopsy findings are rarely reported (10, 15). The patient underwent a relatively lengthy diagnosis and treatment process, during which the symptoms gradually worsened, followed by a large pleural effusion that may suggest a migrating parasite in the pleura. Moxifloxacin treatment alleviated the symptoms but was not curative until an adequate praziquantel dose was administered. To date, there has been no recurrence of clinical symptoms or abnormal chest CT findings at 26 months of follow-up (Follow-up until Dec 2023).

## Data Availability

The original contributions presented in the study are included in the article/supplementary material, further inquiries can be directed to the corresponding author.

## References

[1] Liu Q Li MW Wang ZD Zhao GH Zhu XQ. Human sparganosis, a neglected food borne zoonosis. Lancet Infect Dis. (2015) 15:1226–35. doi: 10.1016/S1473-3099(15)00133-4 26364132

[2] Cheng KB Gao BL Liu JM Xu JF. Pulmonary sparganosis mansoni: a case report from a non-endemic region. J Thorac Dis. (2014) 6:E120–4. doi: 10.3978/j.issn.2072-1439.2014.06.07, PMID: 24977019 PMC4073403

[3] Kim JG Ahn CS Sohn WM Nawa Y Kong Y. Human sparganosis in Korea. J Korean Med Sci. (2018) 33:e273. doi: 10.3346/jkms.2018.33.e273, PMID: 30369856 PMC6200907

[4] Anantaphruti MT Nawa Y Vanvanitchai Y. Human sparganosis in Thailand: an overview. Acta Trop. (2011) 118:171–6. doi: 10.1016/j.actatropica.2011.03.011 21459073

[5] Li N Xiang Y Feng Y Li M Gao BL Li QY. Clinical features of pulmonary sparganosis. JAMA. (2015) 350:436–41. doi: 10.1097/MAJ.0000000000000578, PMID: 26465081

[6] Matsuki M Higashiyama A. Pulmonary sparganosis: tunnel sign and migrating sign on computed tomography. Intern Med. (2020) 60:601–4. doi: 10.2169/internalmedicine.5304-20, PMID: 32999230 PMC7946513

[7] Chen JX Cai YC Liu Ai L Song P Chen SH Chen SH. Epidemic status and challenges of important human parasitic diseases in China. Lab Med. (2021) 36:993–1000. doi: 10.3969/j.issn.1673-8640.2021.010.001. (in Chinese).

[8] Tong DS. Current status of epidemiological investigation and research on sparganosis mansoni in my country. Chin J Sch Doctor. (2018) 32:395. (in Chinese).

[9] Chen X Bai J Wang J Cheng K Shen CM Yao H. Sparganosis presenting as pericardial effusion and lung lesions. Intern Med. (2015) 54:1135–9. doi: 10.2169/internalmedicine.54.3478, PMID: 25948364

[10] Li H Hu J Yang P. Diagnosis and treatment of human sparganosis. Lancet Infect Dis. (2019) 19:577–8. doi: 10.1016/S1473-3099(19)30218-X 31122772

[11] Sivakorn C Promthong K Dekumyoy P Viriyavejakul P Ampawong S Pakdee W. Case report: the first direct evidence of *Gnathostoma spinigerum* migration through human lung. Am J Trop Med Hyg. (2020) 103:1129–34. doi: 10.4269/ajtmh.20-0236, PMID: 32588815 PMC7470552

[12] Keerthana VV Azeez AK Niyas VKM Abraham SV Rajeswari RA Abraham R. A curious case of pulmonary anisakiasis. Pulmon. (2024) 26:66–8. doi: 10.4103/pulmon.pulmon_16_24

[13] Miyamoto N Mishima K Nagatomo KI Ishikawa N Ohashi T Eto T. A case report of serologically diagnosed pulmonary gnathostomiasis. Jap J Parasitol. (1994) 43:397–400.

[14] Chung SW Kim YH Lee EJ Kim DH Kim GY. Two cases of pulmonary and pleural sparganosis confirmed by tissue biopsy and immunoserology. Braz J Infect Dis. (2012) 16:200–3. doi: 10.1016/S1413-8670(12)70307-0, PMID: 22552467

[15] Bai J He ZY Liu GN Zhang JQ Deng JM Li MH. Bronchial *Sparganosis mansoni* accompanied by abnormal hyperplasia diagnosed by bronchoscopy. Chin Med J. (2012) 125:3183–7. doi: 10.3760/cma.j.issn.0366-6999.2012.17.041, PMID: 22932205

